# Extract from a Series of Observations Communicated to the Society of Medicine at Paris

**Published:** 1799-08

**Authors:** Michael Christopher Lombart

**Affiliations:** First Surgeon at Rethel


### *Extract from a Series of Obfervations communicated to the Society of Medicine at Paris: By Cit.* Michael Christopher Lombart, *Firft Surgeon at Rethel.*

Though feveral fimilar obfervations are to be found in the works of art, the fociety were of opinion, that the following ought to be publifhed, becaufe medical facts, in general, prefent particular fhades, which in fome degree differ from each other. We fometimes alfo meet with complications, which render an operation either very difficult or unfuccefsful, if the furgeon does not inftantly diftinguifh, whether he ought to avail himfelf of the refources of art or nature. It muft be acknowledged that Citizen Lombart has properly attended to the indications which occurred in the cafes of which he gives us an account.

Observation I. *On the good Effects of Compreffes moiftened with cold Water, and applied immediately to tumefied Inteftines, in the operation for Hernia.*

A woman, thirty-five years of age, fuffered during thirteen days from a ftrangulated abdominal hernia, fituated two inches below the umbilicus, and laterally one inch from the *linea alba*; this hernia was of long ftanding, and fucceeded an abfcefs which took place in the epigaftric region. Every means had been unfuccefsfully tried, to affuage the pain and reduce the parts. A quack, who was confulted as the laft refource, applied to the tumour a heating amulet, which increafed the complication of fymptoms. The tumour, which was of an extraordinary fize, reprefented the form of a hat; its furface was covered with *phlyctænæ*; a perpetual hiccup greatly tormented the patient. Such was the ftate of things, when, on the twelfth day, Citizen Lombart was called in. Finding the patient in a defperate ftate, he wifhed to decline his his aſſiſtance, when the patient earneſtly requeſted that the operation for hernia might be performed; which he determined to comply with on the following night, being the thirteenth of the diſeaſe. He firſt made an inciſion from the xiphoid cartilage to the umbilicus, and was obliged to make the crucial inciſion to diſcover the inteſtinal ſac; the inteſtines were extremely inflated; the abdomnal iaperture through which they paſſed, much diſtorted; the edges rather callous; he made inciſions above and below, to leſſen the contraction; but with all his attempts he could not ſucceed in reducing the inteſtinal ſac.

Citizen Lombart remained in uncertainty how to accompliſh the reduction, when the patient aſked for a glaſs of cold water to allay the hiccup, which was increaſing, and did not a little impede its reduction. This demand of the patient ſtruck the operator with the idea, of applying to the inteſtinal ſac compreſſes moiſtened with cold water; notwithſtanding the length of time the ſtrangulation had continued, there was not the leaſt change diſcoverable in the inteſtines. A few moments after this application, a borborygmus announced a diſpoſition in the parts to return to their proper ſituation. In fact, this reduction was made with great facility, the patient's wound was dreſſed, and ſhe was confined to the care of Citizen Guntelot; the ſymptoms in a ſhort time abated. Ten days after, an abſceſs took place at one of the corners of the wound, the cauſe of which was attributed to the ſtimulating amulet which had been applied a few days before the operation. The tumour was afterwards opened, ſince which the patient has gradually got better, and, on the twenty-ſeventh day after the operation, was completely cured.

Observation II. *Diſtortion of the abdominal Viſcera, and particularly of the Liver, occaſioned by the long continued uſe of Whalebone Stays.*

The female Citizen Remont, twenty years of age, wore from her infancy, according to the cuſtom then prevalent, a pair of light whalebone ſtays; ſhe conſtantly experienced inſupportable pains and uneaſineſs. In vain ſhe complained of the diſagreeable ſenſations ſhe experienced; her parents paid no attention to her diſtreſs, and ſhe was compelled to be thus encaſed till ſhe was nineteen years old, at which time ſhe was married. Her ſhape, neverthleſs, was not in the leaſt deformed. Wiſhing to take advantage of the liberty which her new ſtate afforded her, ſhe laid aſide her whalebone ſtays, but ſoon became ſubject to ſuch violent fainting fits, that ſhe was incapable of taking the leaſt exerciſe; which ſymptoms ſhe was ſubject to almoſt every evening when ſhe undreſſed herſelf; they had continued for ſeveral years, the cauſe of which was attributed to the conſtraint ſhe had been under in the day time, by being obliged to wear tight-laced ſtays.

A phy-

A physician who was consulted, attributed the cause to the sudden change of her confined dress. He advised the young lady to loosen her stays by degrees: she followed his advice.

Notwithstanding these precautions, she continually experienced a dull pain in the right hypochondrium, which was attributed to the vapours: she had three children, and attained the age of eighty years.

When she was seventy-nine years old, her pains became insupportable, and changed their place. A most voluminous tumour settled in the region of the left iliac, the contents of which appeared schirrous: baths, emollients, and solvents were administered: the disease did not make less progress in her bowels. At this time I was consulted, adds Citizen Lombart, and after the recital I heard from the patient, of what she had suffered in the course of her life, through tight clothes, I imagined that the viscera, having been continually in a constrained state, particularly before she arrived at the age of nineteen, might possibly be displaced. I suspected the liver to be the seat of the tumour, and communicated this idea to my partner, who observed, I was mistaken, as the liver is generally situated in the right hypochondrium, and the tumour appeared in the left iliac region; which, besides, the patient not having an icteric complexion, one could not reasonably suppose this viscus affected. From that time I did not see the patient, who became yellow in a few days, and shortly after died. I was requested to open her, which I performed in presence of the family-physician of the deceased, and my friend Telinge, correspondent of the Society.

On opening the abdomen, we found the region usually occupied by the liver deprived of its appendages: the ligaments, which were entangled, and not distinguishable, had formed adhesions, the diaphragm appeared depressed, the liver had descended into the pit of the left iliac, and adhered to the viscera; the gall bladder was empty, its partitions met together, the cistic and cholidoc canals apparently obliterated, the duodenum had changed its place, the peritonæum was tuberculous, and intimately connected with the liver; the whole presented an ununiform mass. An incision into this tumour caused a great quantity of pus to be discharged. Having introduced my finger into the opening, I withdrew from thence a very large stone. The whole mass having been separated, I divided it into two parts: we discovered a capsule in which were contained nine biliary calculi, of a cubic form, and different sizes. The fluid was extremely thick, and presented the shape of a gizzard; its internal surface was of a white colour.

Observation III. *Abscess of the Liver.*

A woman of Bertoncourt, near Rethel, was suddenly attacked with dull pains in the right hypochondrium, and remained two years in this suffering state, without cessation from her rural labours; the pains then became so acute, acute, that ſhe was obliged to keep at home; a ſurgeon who was called in, diſcovered a tumour, on which he ordered emollients to be applied; the fluctuation being manifeſt, he made an inciſion, which cauſed a diſcharge of a great deal of pus and ſome biliary calculi; on the following days the patient diſcharged, at different times, a great number of concretions of different ſizes; the wound ſoon after cicatrized, and the woman was able to reſume her cuſtomary occupations.

Six years after, the complaint returned, but with leſs ſeverity; the ſurgeon who had formerly attended her being dead, I was conſulted. I found a tumour of the ſize of a hen's egg; I opened it, and a ſmall quantity of ſerous pus was diſcharged, together with a biliary calculus of the ſize and form of a French bean. I uſed detergent injections; ſome time after, the ulcer partly cicatrized; but there ſtill remained a little fiſtulous hole, which at various intervals diſcharged matter. At length, being aſſured that no foreign body remained in it, I cauteriſed the fiſtula with the lunar cauſtic, ſome days after which, the cure was completed.

Observation IV. *Account of the diſcharge of an Inſect from the Noſe.*

Citizen Charles B****, was afflicted in his infancy with violent pains in his head, which were often accompanied with convulſive and debilitating ſymptoms. Theſe diſorders were attributed to the preſence of worms in the ſtomach, or inteſtines. Vermifuges were preſcribed, but without affording any relief. Paroxyſms of pain took place at different intervals. The growth of the patient was nevertheleſs not retarded. When he attained the age of puberty, his parents deſigned him for the church, but he was not admitted to the degrees, becauſe he was conſidered as epileptic, on account of the frequent recurrence of violent ſpaſms, to which he was ſubject, at various times. He preferred the army, and from this new employment ſeemed to derive real benefit; till the age of thirty-two, he did not experience any violent criſis, but then became in ſome degree familiariſed to his complaint. At the ſame time he laboured under ſo ſevere a nervous diſorder, that a door ſuddenly opened, or ſhut with force, occaſioned the moſt violent ſpaſms; his mind was ſo much affected, that the leaſt anxiety or diſappointment threw him into convulſions. He conſulted me in the year 1790, and informed me of the means he had been adviſed to adopt till the preſent time, all of which he had tried without ſucceſs. I was not more ſucceſsful than my predeceſſors in the directions I gave him.

An old woman adviſed him to take ſnuff, or ſome other ſternutatory; he complied with her advice, and this remedy occaſioned frequent ſneezings, after which he diſcharged from the noſe a living inſect, a kind of caterpillar, of a dirty white colour, which had fifteen rings; its legs were covered with hair. Citizen B. has since been totally relieved from his head-ach, but still remains afflicted with nervous symptoms.

I sent this insect to Vicq d'Azir, who has given it a place in his collection. I have not been informed what use he has made of the observation.

[The French Editor remarks, that he intends to continue the series of observations in his next Number.]

